# Roles of Spatial Scale and Rarity on the Relationship between Butterfly Species Richness and Human Density in South Africa

**DOI:** 10.1371/journal.pone.0124327

**Published:** 2015-04-27

**Authors:** Silvia Mecenero, Res Altwegg, Jonathan F. Colville, Colin M. Beale

**Affiliations:** 1 Department of Zoology and Entomology, University of Pretoria, Pretoria, South Africa; 2 South African National Biodiversity Institute, Kirstenbosch Research Centre, Claremont, South Africa; 3 Department of Biodiversity and Conservation Biology, University of the Western Cape, Belville, South Africa; 4 Statistics in Ecology, Environment and Conservation, Department of Statistical Sciences, University of Cape Town, Rondebosch, South Africa; 5 Department of Biology, University of York, Heslington, York, United Kingdom; Consiglio Nazionale delle Ricerche (CNR), ITALY

## Abstract

Wildlife and humans tend to prefer the same productive environments, yet high human densities often lead to reduced biodiversity. Species richness is often positively correlated with human population density at broad scales, but this correlation could also be caused by unequal sampling effort leading to higher species tallies in areas of dense human activity. We examined the relationships between butterfly species richness and human population density at five spatial resolutions ranging from 2' to 60' across South Africa. We used atlas-type data and spatial interpolation techniques aimed at reducing the effect of unequal spatial sampling. Our results confirm the general positive correlation between total species richness and human population density. Contrary to our expectations, the strength of this positive correlation did not weaken at finer spatial resolutions. The patterns observed using total species richness were driven mostly by common species. The richness of threatened and restricted range species was not correlated to human population density. None of the correlations we examined were particularly strong, with much unexplained variance remaining, suggesting that the overlap between butterflies and humans is not strong compared to other factors not accounted for in our analyses. Special consideration needs to be made regarding conservation goals and variables used when investigating the overlap between species and humans for biodiversity conservation.

## Introduction

Conserving biodiversity is important for maintaining ecosystem services [1, 2] and stability [3, 4], which are essential to human welfare. The growth of the human population and activities associated with it are having increasingly negative impacts on biodiversity through habitat loss [5, 6], with a consequent increase in the rate of extinction of species [5, 7]. The extinction crisis may be exacerbated if, as suggested by some authors e.g. [8, 9], wildlife species generally share preferences with humans for the same productive environments. Understanding the relationships between patterns of biodiversity and human impact is necessary for resolving conflicts between biodiversity conservation and activities associated with the growing human population [8].

Species diversity tends to be greater in areas of higher productivity where available energy and water is greatest [10, 11]. Human population density also tends to be highest in more productive areas where resources are more readily available for use [12, 13]. For vertebrates, invertebrates and plants, studies conducted at coarse spatial resolutions have found positive correlations between species richness and human population density [14–19]. At finer spatial resolutions (<10 km^2^), however, this correlation tends to weaken or become negative [14, 18, 20, 21]. This is generally assumed to be evidence that broadly similar abiotic processes drive large-scale patterns of both wildlife and human populations, but coexistence is less likely at finer scales [22]. Where positive correlations are observed, conservation of biodiversity in densely populated areas becomes more challenging due to conflicts between human development and species requirements [8, 12]. Although total species richness is often driven by widespread generalist species of little conservation value [23], a similar relationship has been reported for threatened and range-restricted vertebrate species e.g. [8, 24] suggesting real conflicts may exist. Nevertheless, negative impacts of humans may not always correspond with high human densities as human impacts may extend beyond areas of high population density, such as agricultural and forestry developments [5].

Further uncertainty in the potential conflict between species richness and human population density arises from the study bias towards vertebrates and coarse spatial resolutions (units of 2 500 km^2^ and larger) [15]. Studies based on invertebrates are notably scarce [14–19, 25, 26] as are studies conducted at finer spatial resolutions [20, 24, 27]. The value of using invertebrates to study biodiversity patterns and processes is being increasingly recognised [28] due to the pivotal role they play in providing ecosystem services [29] and because they comprise the bulk of biodiversity.

Another major problem for understanding the relationship between human population density and biodiversity across large areas in general is that true species richness is unknown due to species going undetected and not all places being sampled with equal effort. Sampling is typically more intense in easily accessible areas [15, 30] and the observation process likely correlates with human population density [19, 31]. It is therefore critical that in cases where it is logistically difficult to conduct rigorous surveys, as with butterflies in South Africa, true species distributions are first estimated. Ideally, species richness measures should take into account the uncertainties arising from the observation process.

Here we use butterflies, a flagship group for insect conservation [32], to investigate the relationship between species richness and human population densities. Butterflies have been under-represented in large-scale spatiotemporal insect studies globally e.g. [14, 16, 33], especially in South Africa [34, 35]. This is notwithstanding that South Africa is an important area for butterfly biodiversity, containing about 17% of species in sub-Saharan Africa [36], of which half are endemic to South Africa and 19% are of high conservation priority [23]. A recently completed national butterfly atlasing project [30] gave us the opportunity to use a comprehensive butterfly distribution database, in conjunction with geostatistical modeling, to assess, at both coarse and fine spatial resolutions, the relationship between species richness and human population density. Specifically, we tested the following hypotheses: 1) the correlation between total butterfly species richness and human density is positive at each spatial resolution, 2) the positive correlation is weaker at finer spatial resolutions, 3) any correlation that exists is primarily driven by common species, 4) the correlation between richness of threatened or restricted range species and human population density is also positive, and 5) land transformation reduces richness but only once the effect of human population density is accounted for.

## Methods

### Data

**Fig 1:**
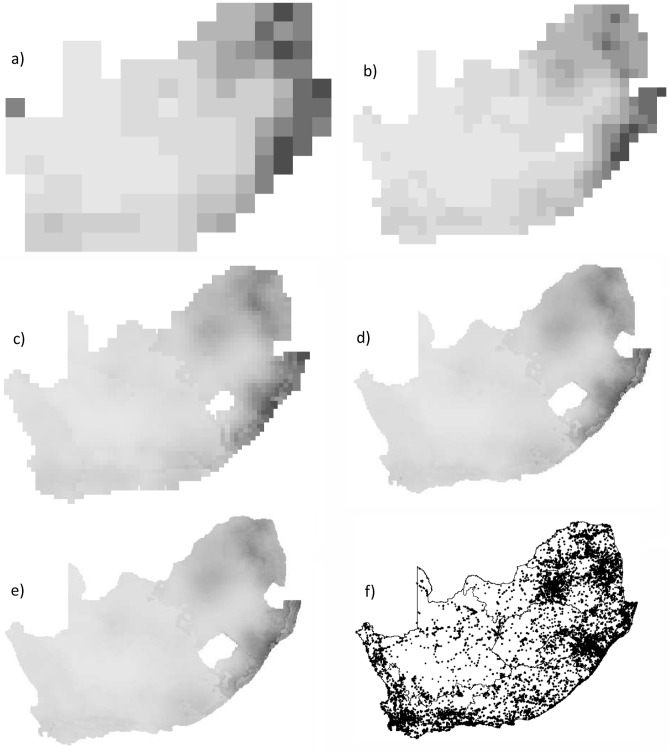
Modelled median total richness for South African butterflies at five grid square scales. Modelled median total richness (Spatial Model 1) for butterflies in South Africa at five grid square scales: a) 60 minutes, b) 30 minutes, c) 15 minutes, d) 5 minutes and e) 2 minutes. Higher richness is represented by darker shades of grey. In f), point localities of all butterfly distribution records (*n* = 326 530) in the atlas region (South Africa, Lesotho and Swaziland) which emanated from the SABCA project [30] are shown.

We obtained butterfly distribution data from the Southern African Butterfly Conservation Assessment project (SABCA; see [30] for details). To interpolate the butterfly distributions for South Africa, we included distribution data from Lesotho and Swaziland (the atlas region) and then extracted the interpolated probabilities to determine richness for South Africa only. Distribution data were available for all 657 recognised species of butterflies in the atlas region (all of which occur in South Africa) and there were a total of 326 530 distribution records (Fig 1F). The SABCA project determined the global Red List status of all the butterfly species and subspecies in the atlas region according to the IUCN system [30, 37]. Here, we adapted the Red Listing to species only, using the same Red Listing system. Of the 657 species, 39 (5.9%) are threatened (eight Critically Endangered, 19 Endangered and 12 Vulnerable). Restricted range species were defined as those with a range less than 500 km^2^ and 50 species (7.6%) were classified as such. Of these, 24 are threatened species.

Environmental data for the entire atlas region were obtained from [38]. Of the eight environmental variables originally chosen as the most relevant to butterfly biology, we selected five for spatial modeling following collinearity tests. Collinearity tests included Pearson’s correlation coefficients (R) with pair-wise plots, coefficients of determination (unadjusted R^2^) and variance inflation factors [39]. The five chosen variables were: mean summer temperature (°C, mean of January daily maximum temperatures), mean winter temperature (°C, mean of July daily minimum temperatures), mean annual precipitation (mm), rainfall concentration (ranges between 0% for zero seasonality to 100% for all rainfall in a single month) and rainfall seasonality (a categorical variable with six categories each representing a type of rainfall seasonality: all year, winter, early summer, mid-summer, late summer, very late summer; all numerical covariates had correlations below 0.68). Environmental data were prepared at the one minute grid square scale using ESRI ArcMap 10.0.

We obtained data for the 2001 South African human population census from Statistics South Africa (http://www.statssa.gov.za). These data were available as counts in 56 255 polygons representing administrative units. ESRI ArcMap 10.0 was used to convert these data to a one minute grid of average human population density (number of people per 1 km^2^).

The proportion of land transformed in South Africa was used as a measure of the impact of human activities. We obtained land cover data from the South African National Biodiversity Institute’s Biodiversity GIS data portal [40]. Data were classified into seven land cover types, two of which were natural classes (natural and water bodies) and five were transformed classes (cultivation, degraded, urban built-up, plantations and mines). The proportion of transformed habitat within each one minute grid square was determined using ESRI ArcMap 10.0 and the Geospatial Modeling Environment tool [41].

Data for human population density and proportion land transformed were available only for South Africa and not the entire atlas region, thus further analyses used the extent of South Africa only. Grid squares which extended beyond South Africa (i.e. into the ocean or neighbouring countries) were given the species richness and anthropogenic values corresponding to the section of the grid square which fell within the study area. Anthropogenic values were prepared at the one minute grid square scale, and were aggregated (mean of the values) to each of the five grid square scales employed in this study. Log human population density was positively correlated to the logit of proportion land transformed at each spatial resolution: 60 minutes (r = 0.86), 30 minutes (r = 0.81), 15 minutes (r = 0.76), 5 minutes (r = 0.71) and 2 minutes (r = 0.65).

We ran all models and analyses using the program R (version 2.15.0; [42]) within the RStudio integrated development environment (version 0.95.265). Models were run on a powerful server machine with dual processors to handle the intensive modeling (2 x 1 TB hard drives, 32 GB RAM, 64-bit operating system, 2 x XEON 2.13 GHz processors giving eight processing cores).

### Spatial modeling

One of the limitations associated with atlas-type and presence-only data is that observer effort is biased towards those areas that are most easily accessible and to those species of special interest or better detectability [15, 18, 30]. In an effort to overcome these biases, we employed geo-spatial modelling to determine species richness.

We developed a model (Spatial Model 1) to spatially interpolate the distribution records for each butterfly species and to obtain a probability of occurrence for each species. The model used regression kriging for the final interpolation of recorded presence and is analogous to the method proposed by [43]. The probabilities were summed across species as a measure of species richness. Then we related species richness to human population density and land use change. We repeated the above at five different spatial resolutions. The main steps are described below and outlined in Table 1.

**Table 1:** Steps used for spatial distribution modelling and species richness determination, using Spatial Models 1.

*Step 1: Prepare data*
• Identify locations (distribution records) where species X is present.
• Identify flight months for species X.
• Identify localities of all other species which: a) >100 m from those of species X and b) sampled in the flight season of species X.
• Randomly select pseudo-absences from of these points, based on geographical and environmental distance, so that the sum of the number of presences and number of pseudo-absences was 1 700.
• Geographical distance probability surface: a) calculate density surface of true presences for species X; b) calculate density surface for localities of all species; c) subtract density surface for species X from that for all species.
• Environmental distance probability surface: a) conduct principle component analysis on numerical co-variates; b) determine environmental range of species X; c) determine localities for all species which are beyond 10% of this range.
• For each grid square select the maximum of the geographical and environmental probability = probability of absence surface from which pseudo-absences are selected.
• Repeat pseudo-absence selection procedure nine times = nine probability realisations.
*Step 2: Spatial interpolation of distribution records*
• Conduct regression kriging for each species with >5 distribution records to obtain an estimate of the occurrence probability with standard error.
• Repeat for each of the nine pseudo-absence sets.
• Take 10 random draws of probability values with mean and variance from the regression kriging results = 10 probability realisations.
• For species with <5 distribution records set occurrence probability to one in grid cells where the species was detected.
*Step 3: Estimate species richness*
• For each of the 90 probability realisations (10 random draws for each of the nine pseudo-absence sets), sum the probabilities across all species to obtain 90 different species richness realisations.
*Step 4: Assess sensitivity of results to pseudo-absence selection*
• Steps 1–3 above were repeated in the form of a second model (Spatial Model 2), to determine if the manner in which pseudo-absences were selected in Spatial Model 1 had an effect on the outcomes. Spatial Model 2 differed from Spatial Model 1 by selecting pseudo-absences in the following way: pseudo-absences were selected as all of the localities identified for all other species which did not contain a record for the focal species.
• Step 3 resulted in 10 species richness realisations (one pseudo-absence set and 10 random draws).

Species X reflects the focal species being modelled. The steps were carried out at five spatial resolutions. The steps were repeated using Spatial Model 2 (Step 4).

#### Step 1: Prepare data

All records for a particular species were selected from the database. From these records information on the months that adult butterflies are in flight was extracted. With presence-only data, one has to make assumptions about the unobserved absences. We chose to select pseudo-absences in a way that accounted for sampling intensity and the likelihood of a species to be absent, based on its proximity to observed occurrences and environmental conditions. Localities of all other species were identified which a) were at least 100 m from records of the focal species and b) had been sampled during the flight period of the focal species. From these localities we randomly selected pseudo-absences, with probabilities that depended on the geographical and environmental distance away from known localities of the focal species, as described below. The number of pseudo-absences selected depended on the number of presences, so that the sum of both was 1 700.

For geographical distance, we computed a 2-d kernel density estimate using the presence data of the focal species, with the standard deviation for the smoothing set by confidence scores (high confidence set small standard deviations) derived by lepidopterist experts. An approximate confidence score from 1 to 5 was ascribed to these records: 5 indicated a strong *a priori* belief that all true populations were accurately represented in the data; 1 indicated a belief that real populations were highly likely to be missing from the data, even far from known records. This density was subtracted from an equivalent density surface for presence data of all species (indicative of observer effort), to produce a geographical distance probability surface on which we based pseudo-absence selection. For environmental distance, we undertook a principle component analysis on the four numerical co-variates and first calculated the centroid (in environmental space) of the known presences and then the environmental distance of each cell from this centroid. We scaled environmental distance from 1 to 0 and set as zero all cells with environmental distances within the environmental space spanned by known presences, plus 10% on all environmental distances. Geographical and environmental distance probability surfaces were compared and the maximum probability per grid square was selected to produce a final probability of absence surface, from which the subset of pseudo-absences was selected. The pseudo-absence selection process was repeated nine times to investigate the effect of using different subsets of pseudo-absences.

#### Step 2: Spatial interpolation of distribution records

Regression kriging [44] followed the selection of pseudo-absences and was run for each of the nine different subsets of pseudo-absences. Regression kriging is a two-phase process involving fitting an initial, non-spatial, regression model to the presences and pseudo-absences and potentially important spatial covariates (in our case climate data), and then interpolating (using kriging) the residuals of this model to improve the spatial predictions (which we term a 'probability realisation'). We used a general additive model with two knots fitted to the five environmental variables in the regression step [45]. Regression kriging generates an estimate of the mean and variance in the probability of presence in each grid cell.

To assess uncertainty in these surfaces, we made 10 random draws of interpolated probability values with appropriate mean and variance from the regression kriging results. This created 10 'probability realisations' for each species. Overall, for each species modelled there were 90 probability realisations (nine pseudo-absence subsets with 10 random draws each).

Steps 1 and 2 could not be run for six species which had fewer than five records. For these six species, we set probabilities for those grid squares that contained distribution records to one.

#### Step 3: Estimate species richness

Within each of the 90 realisations, we summed probability surfaces for all species to obtain species richness. This resulted in 90 species richness realisations [46]. To assess the sensitivity of richness patterns to the nine pseudo-absence subsets used, pair-wise correlations between matching grid squares for the nine richness realisations were determined, within each of the 10 draws. All correlations ranged between 0.99 and 1, indicating that pseudo-absence selection had negligible impact on the overall estimated richness patterns at each spatial resolution.

To determine whether the 10 random draws from the regression kriged surfaces produced different species richness patterns, we carried out pair-wise correlations between matching grid squares for the 10 richness realisations (but within each of the nine pseudo-absence sets). All correlations ranged between 0.99 and 1, showing again that different draws did not affect overall species richness at each spatial resolution. As a result, all further analyses made use of the median of the 90 species richness realisations.

#### Step 4: Assess sensitivity of pseudo-absence selection

In order to determine whether the spatial modelling employed above (Spatial Model 1) is sensitive to the manner in which pseudo-absences were selected (biased by geographical and environmental distance), we developed a second spatial model (Spatial Model 2) with a different approach to pseudo-absence selection. We repeated steps 1 to 3 above, but with the following differences: a) all localities identified for all other species were used as pseudo-absences, thus biasing towards areas with the greatest number of observations; b) there was only one pseudo-absence set (compared to the nine in Spatial Model 1) thus ultimately there were 10 probability realisations per species modelled (one pseudo-absence set and 10 random draws); c) step 3 produced 10 species richness realisations; and d) as with Spatial Model 1, pair-wise correlations used to determine whether the 10 random draws from the regression kriged surfaces produced different species richness patterns, showed that all correlations ranged between 0.99 and 1, thus all further analyses made use of the median of the 10 species richness realisations.

#### Step 5: Compare spatial models

To test the difference in total species richness results between Spatial Models 1 and 2, we correlated the median richness values in matching grid squares between the two models. Correlation between the two models was high, ranging between 0.89 and 0.95 (see S1 Fig), indicating that the richness results are qualitatively similar. All further analyses presented in the main text were thus based on the median species richness values determined using Spatial Model 1 (see S1 Supporting Information for analyses using species richness values of Spatial Model 2).

We carried out the above steps at five spatial resolutions: 60 minute (154 grid squares; ~ 100 km x 100 km), 30 minute (553 grid squares; ~ 50 km x 50 km), 15 minute (2 082 grid squares; ~ 25 km x 25 km), five minute (17 877 grid squares; ~ 8.3 km x 8.3 km) and two minute (110 115 grid squares; ~ 3.3 km x 3.3 km) grid squares.

### Relating species richness to human density and land use

We used linear modeling to examine the hypotheses at each spatial resolution. Four linear models were run at each grid square scale: (a) total species richness ~ log human population density (linear model 1); (b) total species richness ~ log human population density + (log human population density)^2^ (linear model 2); (c) total species richness ~ log human population density + logit proportion land transformed (linear model 3); (d) total species richness ~ logit proportion land transformed (linear model 4). We used the logarithm of human population density to reduce the effect of very high population density in a few grid squares and because we considered that as density increased the incremental impact of additional people reduces. Before logging zero values of human population densities, 0.00001 was added. The coefficient of determination (R^2^) was used to determine the amount of variance explained by the predictor variable(s) in each linear model, and thus the strength of the correlations. The Akaike information criterion (AIC) was used to select which linear model performed the best at each spatial resolution. For comparisons between spatial resolutions, median richness values were centred to mean of zero and scaled to one standard deviation. As our primary interest is in the overall spatial overlap between human impacts and species richness (not the processes that generate this overlap), we were less interested in regression parameters than overall R^2^ and therefore did not need to account for spatial autocorrelation [47].

The linear model which performed the best at each spatial resolution was run again using richness of 25% of the most prevalent, widespread species (which we shall refer to as common species) and 25% of the least prevalent, range-restricted species (which we shall refer to as rare species), based on the quartile definition of species rarity of [48], to determine how these patterns correspond to patterns of overall richness. All four linear models were run to examine the correlations between both threatened (according to IUCN criteria) and restricted range (<500 km^2^) species richness on the one hand, and human population density and land transformation on the other hand.

## Results

Total species richness was highest in the eastern and north-eastern parts of South Africa, followed by the southern and south-western parts, and was lowest in the mid and western regions of the country (Fig 1). Human population density and the proportion of land transformed followed similar trends (see S2 Fig).

When looking at total species richness, linear model 2 (quadratic model of human population density) was the best while linear model 4 (land transformation) had least support at each spatial resolution (Table 2). Linear models 1 and 3 were poorly supported. Subsequently, we used linear model 2 for all further regression analyses involving total species richness.

**Table 2:** Partial regression coefficients (± standard errors; SE), coefficients of determination (R^2^) and AIC values, for four linear models relating total butterfly species richness to human density and activity at five grid square scales (60, 30, 15, 5 and 2 minutes) within the extent of South Africa, using Spatial Model 1: Linear model 1—Total species richness ~ Log human population density (number of people per 1 km^2^); Linear model 2—Total species richness ~ Log human population density + (Log human population density)^2^; Linear model 3—Total species richness ~ Log human population density + Logit proportion land transformed; Linear model 4—Total species richness ~ Logit proportion land transformed.

Grid scale	Linear model	Partial regression coefficient ± SE			Intercept ± SE	R^2^	AIC	Δ AIC
		Log human population density	(Log human population density)^2^	Logit(proportion land transformed)				
60	1	0.23 ± 0.03	-	-	-0.45 ± 0.09	0.26	396.4	15.9
	2	0.07 ± 0.05	0.05 ± 0.01	-	-0.60 ± 0.10	0.34	380.6	0
	3	0.36 ± 0.06	-	-0.13 ± 0.05	-1.06 ± 0.25	0.29	391.3	10.7
	4	-	-	0.12 ± 0.03	0.32 ± 0.10	0.12	423.0	42.5
30	1	0.23 ± 0.01	-	-	-0.40 ± 0.04	0.32	1309.0	33.4
	2	0.15 ± 0.02	0.03 ± 0.01	-	-0.53 ± 0.05	0.36	1275.5	0
	3	0.26 ± 0.02	-	-0.03 ± 0.02	-0.53 ± 0.10	0.32	1308.8	33.3
	4	-	-	0.14 ± 0.01	0.38 ± 0.05	0.18	1406.7	131.1
15	1	0.23 ± 0.01	-	-	-0.29 ± 0.02	0.34	4709.4	80.1
	2	0.17 ± 0.01	0.02 ± 0.003	-	-0.41 ± 0.02	0.37	4629.2	0
	3	0.23 ± 0.01	-	-0.001 ± 0.01	-0.30 ± 0.04	0.34	4711.3	82.1
	4	-	-	0.12 ± 0.01	0.38 ± 0.03	0.20	5102.9	473.7
5	1	0.22 ± 0.003	-	-	-0.16 ± 0.01	0.33	40554.4	467.9
	2	0.20 ± 0.003	0.01 ± 0.001	-	-0.24 ± 0.01	0.34	40086.5	0
	3	0.21 ± 0.004	-	0.01 ± 0.002	-0.11 ± 0.01	0.33	40527.7	441.2
	4	-	-	0.10 ± 0.002	0.37 ± 0.01	0.19	43721.2	3634.6
2	1	0.22 ± 0.001	-	-	-0.10 ± 0.003	0.29	254024.8	3654.0
	2	0.20 ± 0.001	0.01 ± 0.02x10^-2^	-	-0.18 ± 0.003	0.32	250370.8	0
	3	0.20 ± 0.001	-	0.01 ± 0.001	-0.03 ± 0.004	0.29	253643.6	3272.8
	4	-	-	0.07 ± 0.001	0.35 ± 0.004	0.16	272098.2	21727.4

**Fig 2:**
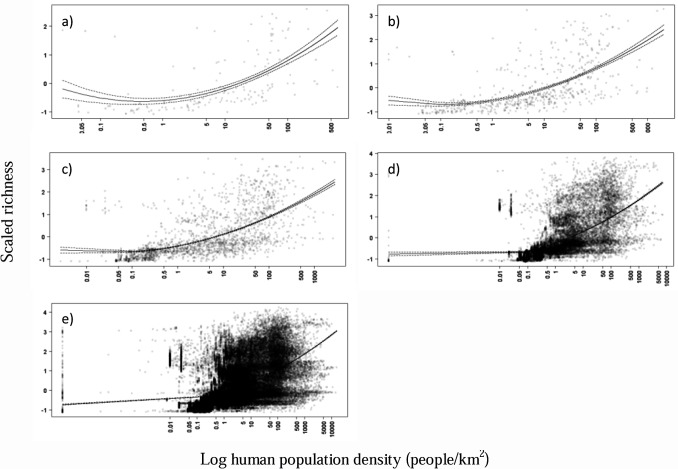
The relationship between butterfly richness and human population density in South Africa, at five spatial resolutions. Scatter plots of modelled median total butterfly richness (scaled and centred) and log human population density, at each of the five spatial resolutions, showing best fitting regression lines (mean ± standard error), for linear model 2 which was the best model in each case: a) 60 minutes, b) 30 minutes, c) 15 minutes, d) 5 minutes and e) 2 minutes.

At all resolutions, linear model 2 described an essentially exponential increase in richness with human density, with very low densities showing universally shallow relationships and higher population densities associated with steeper associations. At coarser resolutions the slopes associated with higher population densities (> 10 people/km^2^) were slightly steeper than the slopes estimated at finer resolutions (Tables 2 and 3, Fig 2). Generally, the hypothesis that the correlation between species richness and human population density is positive is supported.

**Table 3:** Slopes of the quadratic equation of linear model 2, at low (0.01 people per 1 km^2^), medium (10 people per 1 km^2^) and high (500 people per 1 km^2^) levels of population density, based on Spatial Model 1.

Grid square scale	Slope		
	ln(0.01)	ln(10)	ln(500)
60	-0.39	0.30	0.69
30	-0.13	0.29	0.52
15	-0.01	0.26	0.42
5	0.11	0.25	0.32
2	0.11	0.25	0.32

Human population density values were logged.

The strength (R^2^ values) of the positive correlation between total richness and human density did not differ between the spatial resolutions examined, with the coefficients of determination ranging between 0.32 and 0.37 (linear model 2; Table 2). The correlations explained one third of the variance in the data, with about 65% remaining unexplained. Thus the hypothesis that the strength of the correlation weakens with increasing spatial resolution was not supported.

Richness patterns for common species were similar to patterns for total species richness at each spatial resolution (linear model 2; Table 4). In contrast, we found almost no correlation between the richness of rare species and human density. This supports the hypothesis that the correlation between total species richness and human density is driven by common species.

**Table 4:** Partial regression coefficients (± standard errors; SE) and coefficients of determination (R^2^) for linear model 2 (quadratic of log human population density; number of people per 1 km^2^) at five grid square scales (60, 30, 15, 5 and 2 minutes), for common (25% most prevalent) and rare (25% least prevalent) butterfly species in South Africa, using Spatial Model 1.

Species	Grid square scale	Partial regression coefficient ± SE		Intercept ± SE	R^2^
		Log human population density	(Log human population density)^2^		
Common	60	0.11 ± 0.05	0.04 ± 0.01	-0.58 ± 0.10	0.32
	30	0.17 ± 0.02	0.02 ± 0.01	-0.49 ± 0.05	0.34
	15	0.19 ± 0.01	0.02 ± 0.003	-0.36 ± 0.02	0.35
	5	0.21 ± 0.003	0.01 ± 0.001	-0.19 ± 0.01	0.34
	2	0.21 ± 0.001	0.01 ± 0.02x10^-2^	-0.13 ± 0.003	0.30
Rare	60	-0.001 ± 0.05	0.03 ± 0.01	-0.29 ± 0.11	0.08
	30	0.04 ± 0.02	0.01 ± 0.01	-0.18 ± 0.06	0.04
	15	0.07 ± 0.01	0.01 ± 0.003	-0.16 ± 0.03	0.05
	5	0.08 ± 0.003	0.01 ± 0.001	-0.12 ± 0.01	0.06
	2	0.08 ± 0.001	0.01 ± 0.03x10^-2^	-0.09 ± 0.003	0.05

**Fig 3:**
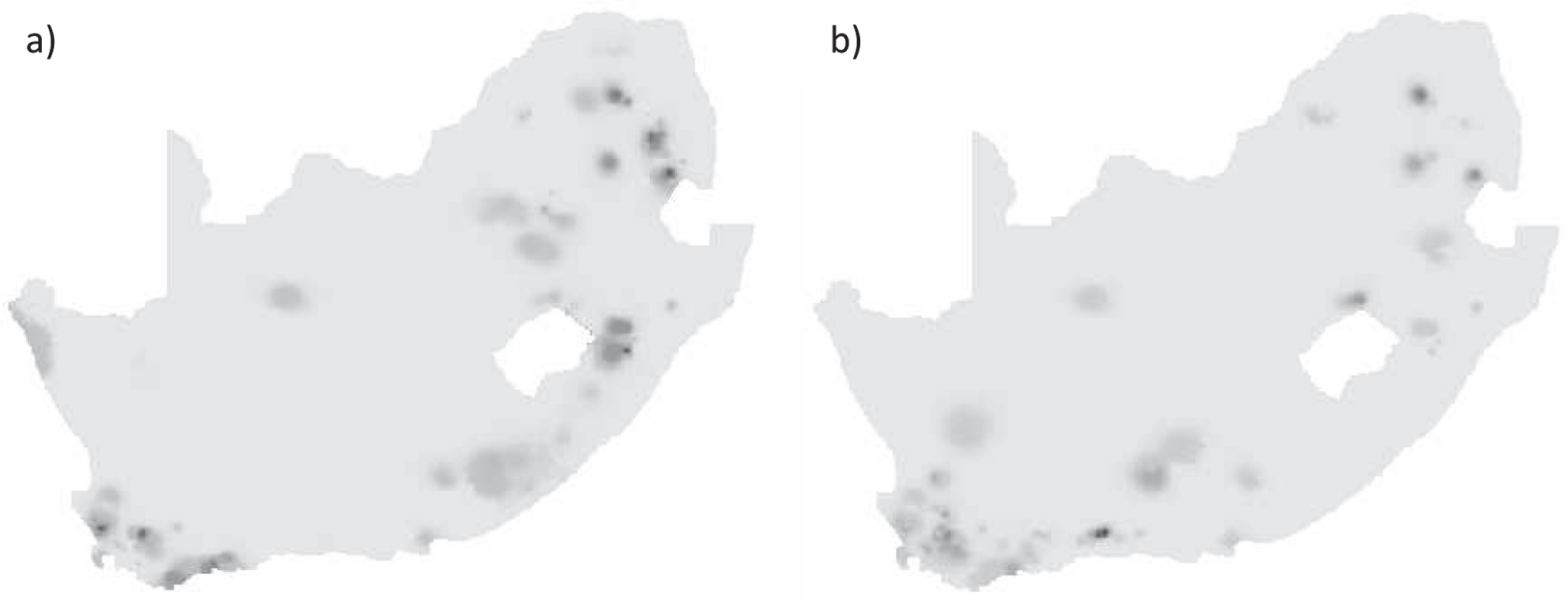
Modelled median richness for threatened and restricted range butterflies in South Africa. Modelled median richness (Spatial Model 1) for (a) threatened and (b) restricted range butterfly species in South Africa at the 2 minute grid square scale. Higher richness is represented by darker shades of grey.

Richness for threatened and restricted range species was patchy with concentrated areas of increased richness (Fig 3). For threatened species richness, linear model 3 performed the best at the coarser spatial resolutions and linear model 4 at the finer spatial resolutions (Table 5). For restricted range species, linear model 1 performed the best at the 60 min grid square unit, linear model 2 at the 30 min grid square unit, and linear model 4 at the finer spatial resolutions. At all spatial resolutions the richness of both threatened and restricted range species were not correlated to human population density (linear models 1, 2 and 3; Tables 5 and 6). The hypothesis that the correlation between richness of threatened or restricted range species and human population density is positive was therefore not supported.

**Table 5:** Partial regression coefficients (± standard errors; SE), coefficients of determination (R^2^) and AIC values, for four linear models relating species richness of threatened butterflies to human density and activity at five grid square scales (60, 30, 15, 5 and 2 minutes) within the extent of South Africa, using spatial Model 1: Linear model 1—Threatened species richness ~ Log human population density (number of people per 1 km^2^); Linear model 2—Threatened species richness ~ Log human population density + (Log human population density)^2^; Linear model 3—Threatened species richness ~ Log human population density + Logit proportion land transformed; Linear model 4—Threatened species richness ~ Logit proportion land transformed.

Grid scale	Linear model	Partial regression coefficient ± SE			Intercept ± SE	R^2^	AIC	Δ AIC
		Log human population density	(Log human population density)^2^	Logit (proportion land transformed)				
60	1	0.003 ± 0.001	-	-	-0.11 ± 0.09	0.06	432.7	0.9
	2	0.01 ± 0.002	-0.04x10^-4^ ± 0.01x10^-3^	-	-0.16 ± 0.10	0.06	433.8	2.1
	3	0.002 ± 0.001	-	0.74 ± 0.43	-0.24 ± 0.11	0.08	431.7	0
	4	-	-	0.03 ± 0.03	0.08 ± 0.11	0.01	440.9	9.2
30	1	0.001 ± 0.03x10^-2^	-	-	-0.04 ± 0.05	0.02	1522.8	39.8
	2	0.004 ± 0.001	-0.02x10^-4^ ± 0.04x10^-5^	-	-0.12 ± 0.05	0.06	1499.8	16.8
	3	0.02x10^-2^ ± 0.03x10^-2^	-	1.36 ± 0.21	-0.29 ± 0.06	0.09	1483.0	0
	4	-	-	0.08 ± 0.01	0.24 ± 0.06	0.06	1496.9	13.9
15	1	0.001 ± 0.01x10^-2^	-	-	-0.02 ± 0.02	0.01	5614.6	202.3
	2	0.002 ± 0.03x10^-2^	-0.01x10^-4^ ± 0.02x10^-5^	-	-0.07 ± 0.02	0.03	5582.1	169.8
	3	-0.01x10^-2^ ± 0.01x10^-2^	-	1.42 ± 0.10	-0.29 ± 0.03	0.11	5412.3	0
	4	-	-	0.09 ± 0.01	0.29 ± 0.03	0.10	5431.1	18.9
5	1	0.03x10^-2^ ± 0.04x10^-3^	-	-	-0.01 ± 0.01	0.003	48026.6	1164.7
	2	0.001 ± 0.01x10^-2^	-0.02x10^-5^ ± 0.02x10^-6^	-	-0.02 ± 0.01	0.01	47953.7	1091.8
	3	-0.03x10^-3^ ± 0.04x10^-3^	-	1.00 ± 0.03	-0.20 ± 0.01	0.07	46929.0	67.1
	4	-	-	0.06 ± 0.002	0.25 ± 0.01	0.07	46861.9	0
2	1	0.02x10^-2^ ± 0.01x10^-3^	-	-	-0.01 ± 0.003	0.002	292983.1	3801.4
	2	0.04x10^-2^ ± 0.02x10^-3^	-0.04x10^-6^ ± 0.03x10^-7^	-	-0.01 ± 0.003	0.003	292844.5	3662.8
	3	0.01x10^-3^ ± 0.01x10^-3^	-	0.61 ± 0.01	-0.12 ± 0.004	0.03	289861.8	680.0
	4	-	-	0.04 ± 0.001	0.19 ± 0.004	0.04	289181.7	0

**Table 6:** Partial regression coefficients (± standard errors; SE), coefficients of determination (R^2^) and AIC values, for four linear models relating species richness of butterflies with restricted ranges in southern Africa to human density and activity at five grid square scales (60, 30, 15, 5 and 2 minutes) within the extent of South Africa, using spatial Model 1: Linear model 1—Restricted range species richness ~ Log human population density (number of people per 1 km^2^); Linear model 2—Restricted range species richness ~ Log human population density + (Log human population density)^2^; Linear model 3—Restricted range species richness ~ Log human population density + Logit proportion land transformed; Linear model 4—Restricted range species richness ~ Logit proportion land transformed.

Grid scale	Linear model	Partial regression coefficient ± SE			Intercept ± SE	R^2^	AIC	Δ AIC
		Log human population density	(Log human population density)^2^	Logit (proportion land transformed)				
60	1	0.001 ± 0.001	-	-	-0.05 ± 0.09	0.01	440.3	0
	2	0.002 ± 0.002	0.01x10^-4^ ± 0.01x10^-3^	-	-0.06 ± 0.10	0.01	442.2	2.0
	3	0.001 ± 0.001	-	0.20 ± 0.45	-0.08 ± 0.12	0.01	442.1	1.8
	4	-	-	0.02 ± 0.03	0.05 ± 0.11	0.003	441.6	1.4
30	1	0.04x10^-2^ ± 0.03x10^-2^	-	-	-0.01 ± 0.05	0.004	1530.8	3.6
	2	0.002 ± 0.001	-0.01x10^-4^ ± 0.01x10^-4^	-	-0.05 ± 0.05	0.01	1527.1	0
	3	0.03x10^-2^ ± 0.03x10^-2^	-	0.26 ± 0.22	-0.06 ± 0.06	0.01	1531.4	4.2
	4	-	-	0.03 ± 0.01	0.10 ± 0.06	0.01	1527.8	0.7
15	1	0.01x10^-2^ ± 0.02x10^-2^	-	-	0.01 ± 0.02	0.001	5669.7	27.6
	2	0.01x10^-2^ ± 0.03x10^-2^	0.04x10^-6^ ± 0.02x10^-5^	-	0.01 ± 0.02	0.001	5671.6	29.5
	3	0.01x10^-3^ ± 0.02x10^-2^	-	0.28 ± 0.10	-0.05 ± 0.03	0.004	5664.3	22.1
	4	-	-	0.03 ± 0.01	0.12 ± 0.03	0.01	5642.1	0
5	1	0.01x10^-2^ ± 0.04x10^-3^	-	-	0.01 ± 0.01	0.04x10^-2^	48316.3	221.7
	2	0.01x10^-2^ ± 0.01x10^-2^	0.01x10^-6^ ± 0.02x10^-6^	-	0.01 ± 0.01	0.03x10^-2^	48317.9	223.3
	3	0.01x10^-3^ ± 0.04x10^-3^	-	0.28 ± 0.03	-0.05 ± 0.01	0.01	48233.5	138.9
	4	-	-	0.03 ± 0.002	0.12 ± 0.01	0.01	48094.6	0
2	1	0.02x10^-3^ ± 0.01x10^-3^	-	-	0.01 ± 0.003	0.02x10^-3^	296352.5	116.4
	2	-0.01x10^-3^ ± 0.02x10^-3^	0	-	0.01 ± 0.003	0.04x10^-3^	296352.3	116.2
	3	-0.04x10^-4^ ± 0.01x10^-3^	-	0.08 ± 0.01	-0.01 ± 0.004	0.001	296298.9	62.8
	4	-	-	0.01 ± 0.001	0.04 ± 0.004	0.001	296236.1	0

For total species richness, there was limited additional benefit of adding proportion of land transformed to the model (linear model 3; Table 2). For threatened species richness, adding the proportion of land transformed showed that land transformation was positively correlated to richness, with the slope of the correlations being highest at the mid-range spatial resolutions and lowest at the 2 min spatial resolution (linear model 3; Tables 5 and 6). However, these correlations were weak (coefficients of determination ≤ 0.11), with at least 89% of the variance remaining unexplained. Restricted range species richness showed no correlation to land transformation. Thus, for total, threatened and restricted range species richness, the hypothesis that the impact of land transformation reduces richness (once shared causes of co-incidence are accounted for) had little or no support at the spatial scales of our analyses.

## Discussion

We examined the relationship between butterfly species richness and human population density across South Africa to quantify the degree to which human activity overlaps with areas of high biodiversity. Specifically, we tested the hypothesis that species richness and human population density are positively correlated at large spatial scales and that this correlation should be weaker at small spatial scales. This pattern could arise if both species richness and human population density were positively correlated with productivity but if human impact reduced species richness locally. Our results confirm the general positive correlation between total species richness and human population density [15], but not the hypothesis that the strength of this correlation should weaken at finer spatial resolutions [18, 20, 22, 27]. In contrast to total species richness, the richness of threatened and restricted range species was not correlated to human population density at any spatial resolution, which is consistent with the contention that biodiversity patterns are driven by common species [23]. None of the correlations we examined were particularly strong with much of the variance remaining unexplained, suggesting that spatial conflict between areas with high butterfly species richness and human density may be relatively limited for butterflies in South Africa.

The positive correlation we observed between total species richness and human population density confirms several other studies [8, 13, 15, 49], including butterfly studies [14, 16]. The primary explanation for this is that both species and humans respond to environments which are most productive [8, 9, 50]. Species rich areas usually coincide with areas of higher primary productivity and higher habitat heterogeneity [5, 11]. Such highly productive areas also attract high human population numbers because of the availability of resources [8, 12, 16, 50, 51]. Primary productivity in South Africa is highest on the eastern side as well as in the southern and south-western parts [38], with similar trends for plant richness [52]. Based on the spatial modeling employed in this study, butterfly species richness in South Africa is highest in the northern and north-eastern parts of the country, followed by the southern and south-western parts. This richness pattern is similar to that for other faunal groups in the region, such as frogs [53], birds [54] and reptiles [55], as well as for flora [56]. Both bird species richness and human population density respond positively to areas of higher primary productivity in South Africa [8]. It is likely that butterflies in South Africa also tend to respond to more productive environments, thus explaining the positive correlation with human population density in our study.

Whereas the strength of the positive correlation between total richness and human density is expected to weaken [15] or become negative [20] at fine spatial resolutions, we found that the strength of the positive correlation remained similar across all spatial resolutions we examined. Although a meta-analysis of studies comparing species richness with human population density found the correlation to weaken as spatial resolution increased, much of the variance typically remains unexplained in such studies [15]. Clearly, there are other drivers of species richness that are (at least in combination) much more important than human population density. One reason why we didn’t find a weakening correlation at finer resolutions could be that our finest resolution used was still too coarse, at about 11 km^2^ (two minute grid square scale) [16]. A study on butterfly richness found that richness began to decrease with level of urbanisation only at the fine scale of 1km^2^ but increased at coarser scales [14]. Similarly, [20] found a negative slope between bird species richness and human density at a 1 km^2^ resolution. Butterflies are small organisms with local-scale requirements [14, 57] and therefore the two minute grid square scale may not have been of sufficient resolution to differentiate between patches with high richness and areas of human density. Alternatively, it is possible that a high number of species are able to persist in or close to high-density human settlements. Human settlements may increase habitat heterogeneity by creating patches where butterflies persist [14, 58], for instance gardens and parks which provide nectar sources for the adult butterflies and host plants for the larvae. Habitat heterogeneity has been found to positively impact butterfly richness [59].

Nevertheless, none of the correlations between total species richness and human population density were strong, with >63% of the variance remaining unexplained in all cases. Although this amount of unexplained variance is typical in similar studies, much tighter correlations are required for generating general rules. A few other studies have found strong correlations where much of the variance was explained (e.g. 81% by [8] and 57% by [33, 60]), however, in these instances the spatial resolution was large (~10 000 km^2^ grid scale or at the level of ecoregions or countries). Studies at finer resolutions (<2 500 km^2^) typically had lower levels of explained variance (<45%; e.g. [20, 24, 61]), while some coarse-scaled studies similarly had low levels of explained variance (e.g. [12, 62, 63]). However, none of these studies commented on the low levels of explained variance and its implication that high richness and human density are not necessarily incompatible.

Studies have found that richness patterns are generally driven by common species [23, 64, 65] and the same seems to be true for butterflies in South Africa. Common species are mainly generalists with a diverse range of host plants and they seem to adapt better to and persist better in transformed environments. Indeed, in the case of our study, areas of high richness of common species were found to overlap with areas of high human population density. The persistence of common species may be because they are able to occupy small untransformed patches within the transformed landscape and/or to exploit transformed habitats [66–68]. Nevertheless, despite common species being able to persist in transformed habitats, it is possible that land transformation can result in a decrease in the population abundance of these species [69], signifying that variables other than richness, e.g. abundance, may be more useful for measuring the impact of overlap between species and humans.

Common species generally consist of species of least conservation concern, compared to the higher conservation needs of threatened and restricted range species which usually have more specialised habitat requirements. Other studies, mainly dominated by birds and mammals and mainly conducted at coarser spatial resolutions (> 2 500 km^2^), show that the richness of threatened and restricted range species is positively correlated to human population density [15]. The fact that we found no clear correlations between the richness of these two categories of species and human population density, and land transformation, at any spatial resolution, suggests that it may be easier to implement conservation measures for these butterflies, especially for those occurring away from human activities. The correlation was not negative, and thus some of these butterflies would still occur within areas of high human density. The placement of protected areas should be balanced between urban and non-urban areas. Particular attention should be given to those occurring in areas impacted by humans. For instance, the Lepidopterists’ Society of Africa (LepSoc) has assisted in the proclamation of four butterfly reserves in South Africa to protect threatened species under threat of urbanisation and habitat degradation (e.g. the Critically Endangered *Orachrysops niobe* and the Endangered *Aloiedes dentatis dentatis*) [70]. LepSoc has recently initiated the Conservation of Rare and Endangered Lepidoptera (COREL) programme to further research and manage threatened butterflies [70]. Nevertheless, our results suggest that habitat patches even in relatively densely populated areas can be important for butterfly conservation (see [71]).

Species richness cannot be observed directly and some species will always go undetected. Unless sampling effort is constant across space, the positive correlation between species richness and human density is therefore always influenced by observer effort, which tends to be higher in areas of higher human activity [15, 18]. This was also true for our study, where observer effort was biased towards areas that are readily accessible to observers from urban or densely populated areas [30]. Even though we employed sophisticated spatial interpolation techniques to reduce possible effects of observer effort, it is possible that such bias was not wholly accounted for. However, our estimated species richness patterns are consistent with expert opinion [72]. For example, areas where observer effort was poor due to low accessibility, such as the northern, north-western and central arid parts of South Africa [30], are known to be species-poor for butterflies based on lepidopterist expert opinion [72] and are species depauperate for a range of other animal groups [73]. Therefore, further sampling would likely not significantly increase the known richness of butterfly species in these areas. Nevertheless, observer bias and measurement of observer effort should be considered in future data collection protocols. Protocols that allow the detection process to be estimated, e.g. through occupancy models, would be preferable [74]. However, we note that the type of data we used is often the only type of data that can be obtained at large spatial scales. Most biogeographical studies are based on this type of data and which have retrieved important patterns of species diversity e.g. [75]. We developed methods that are generally applicable for this kind of data and which will further contribute to obtaining more realistic information on biodiversity patterns at large spatial scales.

Setting priorities for biodiversity conservation depends on the conservation goals, e.g. species diversity versus threatened species [76]. This study found differences between subsets of richness, as have several other studies [18, 67, 77]. Although the correlations in our study were weak when using subsets of richness, these outcomes could influence the type of conservation management strategies to be applied. Therefore, the biodiversity indicators need to be carefully selected when conducting these studies and an understanding using sub-groups of total richness can provide insights into how best to conserve biodiversity in areas of high human impact. Also, considering that total species richness is driven by common species, other ecological and biological variables should be considered when trying to understand the relationship between species richness and human developments.

## Supporting Information

S1 Fig The correlation of butterfly species richness between two spatial models at five spatial resolutions. Scatter plots and correlation coefficients (r) of median butterfly species richness in South Africa between Spatial Model 1 (x-axis) and Spatial Model 2 (y-axis), at each grid square scale: a) 60 minutes, b) 30 minutes, c) 15 minutes, d) 5 minutes and e) 2 minutes. Dashed line indicates slope of one.
(TIF)

S2 Fig South African human population density and proportion land transformed. (a) Human population density (log; number of people/km^2^) and (b) proportion land transformed (logit) in South Africa at the 2 minute grid square scale. High values of human population density and proportion of land transformed are represented by darker shades of grey, as shown by the keys.
(TIF)

S1 Supporting Information Results of relating species richness to human population density using Spatial Model 2. (DOC)
