# Supplementary figures and images for: Roles of Spatial Scale and Rarity on the Relationship between Butterfly Species Richness and Human Density in South Africa
Source: PLoS One. 2015 Apr 27;10(4):e0124327. doi: 10.1371/journal.pone.0124327 (PMC4411036; doi:10.1371/journal.pone.0124327)

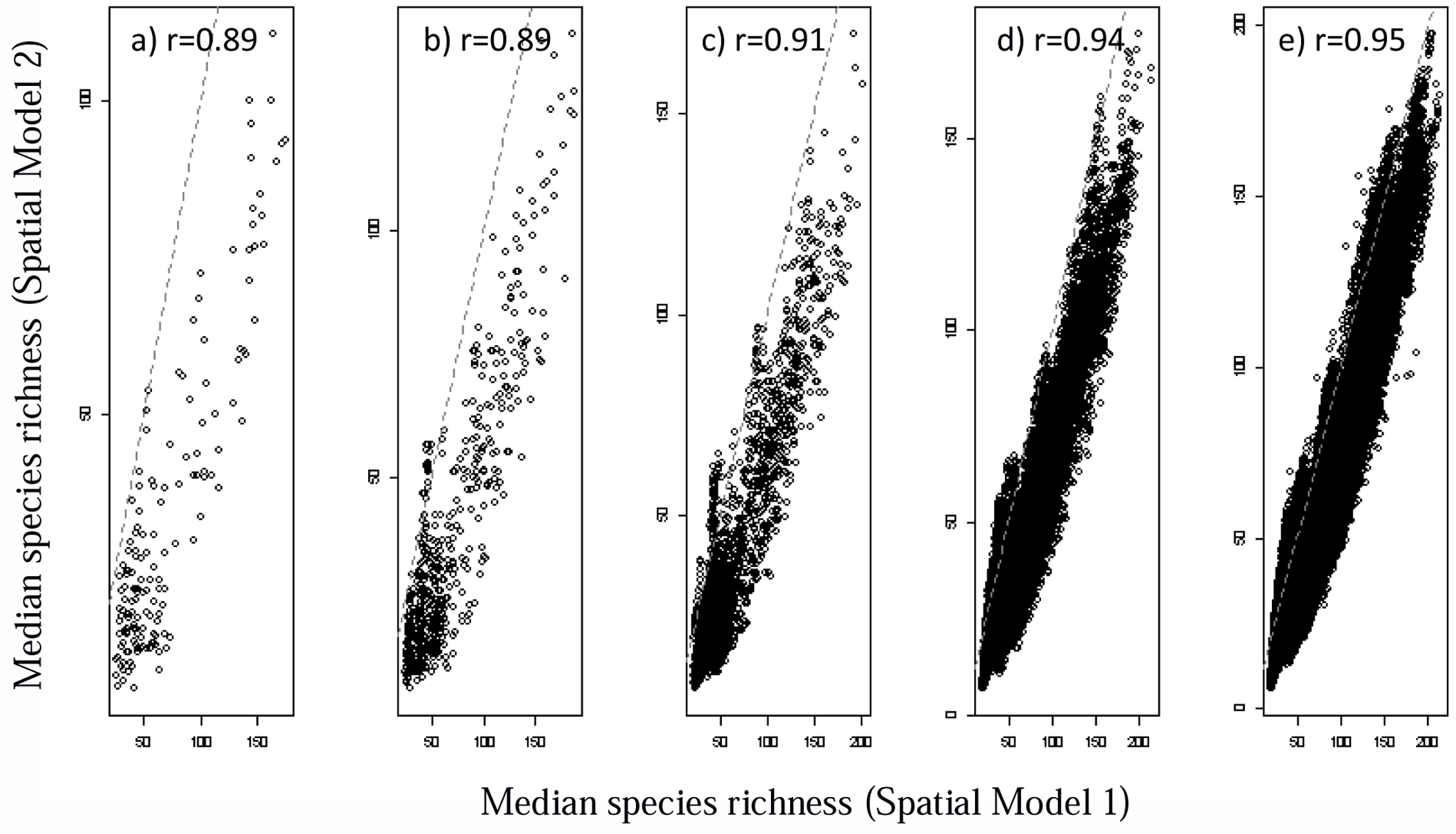

Supplement: S1 Fig — Scatter plots and correlation coefficients (r) of median butterfly species richness in South Africa between Spatial Model 1 (x-axis) and Spatial Model 2 (y-axis), at each grid square scale: a) 60 minutes, b) 30 minutes, c) 15 minutes, d) 5 minutes and e) 2 minutes. Dashed line indicates slope of one. (TIF) [file pone.0124327.s001.tif]

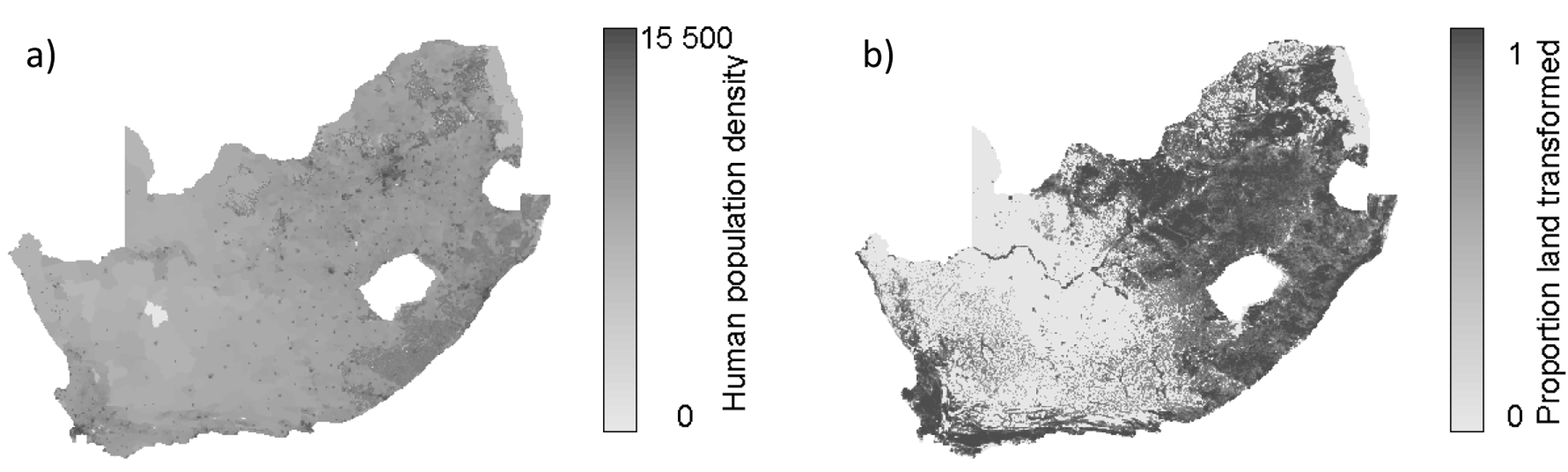

Supplement: S2 Fig — (a) Human population density (log; number of people/km2) and (b) proportion land transformed (logit) in South Africa at the 2 minute grid square scale. High values of human population density and proportion of land transformed are represented by darker shades of grey, as shown by the keys. (TIF) [file pone.0124327.s002.tif]
