# Supplementary material for: Roles of Spatial Scale and Rarity on the Relationship between Butterfly Species Richness and Human Density in South Africa
Source: PLoS One. 2015 Apr 27;10(4):e0124327. doi: 10.1371/journal.pone.0124327 (PMC4411036; doi:10.1371/journal.pone.0124327)
Supplement: S1 Supporting Information — (DOC) [file pone.0124327.s003.doc]

**Supporting information S1**

Median richness values in matching grid squares determined using Spatial Models 1 and 2 were strongly correlated to each other, ranging between 0.89 and 0.95 (Figure S1). Therefore, all further analyses presented in the main text were based on the median species richness values determined using Spatial Model 1.

Human population density and the proportion of land transformed followed similar trends to total species richness; values were highest in the eastern and north-eastern parts of South Africa (Figure S2).

Results for the regression analyses obtained using Spatial Model 1 richness values were very similar to those obtained using Spatial Model 2 richness values (Tables S1-S3). At each spatial resolution, linear model 2 (quadratic model of human population density) was best and linear model 4 (land transformation) had least support. Linear models 1 and 3 performed similarly. The relationship between total species richness and log human population density was generally positive at each of the five spatial resolutions (linear model 2; Tables S1 and S2), in support of the hypothesis. At very low densities relationships were shallow and at higher population densities were associated with steeper associations, while the slopes were steeper at coarser resolutions than at finer resolutions.

**Table S1.** Partial regression coefficients (± standard errors; SE), coefficients of determination (R^2^) and AIC values, for three linear models at five grid square scales (60, 30, 15, 5 and 2 minutes) within the extent of South Africa, using Spatial Model 2: Linear model 1 - Total species richness ~ Log human population density (number of people per 1 km^2^); Linear model 2 - Total species richness ~ Log human population density + (Log human population density)^2^; Linear model 3 - Total species richness ~ Log human population density + Logit proportion land transformed; Linear model 4 - Total species richness ~ Logit proportion land transformed.

a)

| Grid scale | Linear model | Partial regression coefficient ± SE | | | Intercept  ± SE | R^2^ | AIC | Δ AIC | |
| --- | --- | --- | --- | --- | --- | --- | --- | --- | --- |
|  |  | Log human population density | (Log human population density)^2^ | Logit  (proportion land transformed) |  |  |  |  |  |
| 60 | 1 | 0.14 ± 0.03 | - | - | -0.27 ± 0.10 | 0.09 | 426.9 | | 12.1 |
|  | 2 | -0.01 ± 0.05 | 0.05 ± 0.01 | - | -0.42 ± 0.11 | 0.17 | 414.8 | | 0 |
|  | 3 | 0.27 ± 0.07 | - | -0.12 ± 0.05 | -0.88 ± 0.27 | 0.13 | 423.2 | | 8.4 |
|  | 4 | - | - | 0.06 ± 0.03 | 0.16 ± 0.11 | 0.03 | 437.5 | | 22.7 |
| 30 | 1 | 0.17 ± 0.02 | - | - | -0.30 ± 0.05 | 0.17 | 1411.3 | | 21.8 |
|  | 2 | 0.09 ± 0.02 | 0.03 ± 0.01 | - | -0.41 ± 0.05 | 0.20 | 1389.6 | | 0 |
|  | 3 | 0.19 ± 0.03 | - | -0.02 ± 0.02 | -0.40 ± 0.11 | 0.17 | 1412.3 | | 22.7 |
|  | 4 | - | - | 0.10 ± 0.01 | 0.27 ± 0.06 | 0.09 | 1456.8 | | 67.2 |
| 15 | 1 | 0.18 ± 0.01 | - | - | -0.24 ± 0.02 | 0.22 | 5050.8 | | 45.4 |
|  | 2 | 0.13 ± 0.01 | 0.02 ± 0.003 | - | -0.34 ± 0.03 | 0.24 | 5005.3 | | 0 |
|  | 3 | 0.18 ± 0.01 | - | -0.004 ± 0.01 | -0.26 ± 0.04 | 0.22 | 5052.5 | | 47.2 |
|  | 4 | - | - | 0.10 ± 0.01 | 0.29 ± 0.03 | 0.12 | 5275.1 | | 269.8 |
| 5 | 1 | 0.19 ± 0.003 | - | - | -0.14 ± 0.01 | 0.24 | 42540.7 | | 309.2 |
|  | 2 | 0.17 ± 0.003 | 0.01 ± 0.001 | - | -0.21 ± 0.01 | 0.26 | 42231.5 | | 0 |
|  | 3 | 0.18 ± 0.004 | - | 0.01 ± 0.002 | -0.10 ± 0.01 | 0.24 | 42528.2 | | 296.7 |
|  | 4 | - | - | 0.08 ± 0.002 | 0.31 ± 0.01 | 0.14 | 44721.5 | | 2490.0 |
| 2 | 1 | 0.19 ± 0.001 | - | - | -0.09 ± 0.003 | 0.23 | 261771.3 | | 2471.9 |
|  | 2 | 0.18 ± 0.001 | 0.01 ± 0.002 | - | -0.16 ± 0.003 | 0.25 | 259299.3 | | 0 |
|  | 3 | 0.18 ± 0.002 | - | 0.01 ± 0.001 | -0.04 ± 0.005 | 0.23 | 261545.0 | | 2245.7 |
|  | 4 | - | - | 0.06 ± 0.001 | 0.30 ± 0.004 | 0.12 | 275506.6 | | 16207.3 |

**Table S2.** Slopes of the quadratic equation of linear model 2, at low (0.01 people per 1 km^2^), medium (10 people per 1 km^2^) and high (500 people per 1 km^2^) levels of population density, based on Spatial Model 2. Human population density values were logged.

a)

| Grid square scale | Slope | | |
| --- | --- | --- | --- |
|  | ln(0.01) | ln(10) | ln(500) |
| 60 | -0.47 | 0.22 | 0.61 |
| 30 | -0.19 | 0.23 | 0.46 |
| 15 | -0.05 | 0.22 | 0.38 |
| 5 | 0.08 | 0.22 | 0.29 |
| 2 | 0.09 | 0.23 | 0.30 |

**Table S3.** Partial regression coefficients (± standard errors; SE) and coefficients of determination (R^2^) for linear model 2 (quadratic of log human population density; number of people per 1 km^2^) at five grid square scales (60, 30, 15, 5 and 2 minutes), for common (25% most prevalent) and rare (25% least prevalent) species in South Africa, using Spatial Model 2.

| Species | Grid square scale | Partial regression coefficient ± SE | | Intercept  ± SE | R^2^ |
| --- | --- | --- | --- | --- | --- |
|  |  | Log human population density | (Log human population density)^2^ |  |  |
| Common | 60 | 0.01 ± 0.05 | 0.04 ± 0.01 | -0.41 ± 0.11 | 0.16 |
|  | 30 | 0.09 ± 0.02 | 0.02 ± 0.01 | -0.38 ± 0.05 | 0.17 |
|  | 15 | 0.13 ± 0.01 | 0.02 ± 0.003 | -0.31 ± 0.03 | 0.22 |
|  | 5 | 0.17 ± 0.003 | 0.01 ± 0.001 | -0.19 ± 0.01 | 0.24 |
|  | 2 | 0.18 ± 0.001 | 0.01 ± 0.02x10^-2^ | -0.14 ± 0 | 0.24 |
| Rare | 60 | 0.01 ± 0.05 | 0.02 ± 0.01 | -0.24 ± 0.11 | 0.05 |
|  | 30 | 0.07 ± 0.02 | 0.01 ± 0.01 | -0.19 ± 0.06 | 0.05 |
|  | 15 | 0.06 ± 0.01 | 0.003 ± 0.003 | -0.11 ± 0.03 | 0.03 |
|  | 5 | 0.07 ± 0.003 | 0.003 ± 0.001 | -0.09 ± 0.01 | 0.04 |
|  | 2 | 0.07 ± 0.001 | 0.01 ± 0.03x10^-2^ | -0.09 ± 0.003 | 0.04 |

The strength of the positive relationship (R^2^ values) became slightly stronger as the spatial resolution became finer but the coefficients of determination were low and explained not more than one quarter of the variance, ranging from 0.17 to 0.26 with about 75% remaining unexplained (linear model 2; Table S1). The hypothesis that the strength of the relationship weakens with increasing spatial resolution was not supported.

As for spatial Model 1, species richness patterns at each spatial resolution were similar to richness patterns for common species only, thus supporting the hypothesis that the relationship between species richness and human density is driven by common species; we found no relationship between the richness of rare species and human density (linear model 2; Table S3).

For threatened species richness, linear model 2 performed the best at the 60 min grid square unit, whereas linear model 3 performed the best at the finer spatial resolutions (Table S4). For restricted range species, linear model 2 performed the best at most of the spatial resolutions (Table S5). At all spatial resolutions the richness of both threatened and restricted range species were very weakly related to human population density, with coefficients of determination ≤ 0.08 (linear models 1, 2 and 3; Tables S4 and S5). Thus, similar to Spatial Model 1, we could not support the hypothesis that the relationship between richness of threatened or restricted range species and human population density is positive.

**Table S4.** Partial regression coefficients (± standard errors; SE), coefficients of determination (R^2^) and AIC values, for four linear models relating species richness of threatened butterflies to human density and activity at five grid square scales (60, 30, 15, 5 and 2 minutes) within the extent of South Africa, using spatial Model 1: Linear model 1 - Threatened species richness ~ Log human population density (number of people per 1 km^2^); Linear model 2 - Threatened species richness ~ Log human population density + (Log human population density)^2^; Linear model 3 - Threatened species richness ~ Log human population density + Logit proportion land transformed; Linear model 4 - Threatened species richness ~ Logit proportion land transformed.

a)

| Grid scale | Linear model | Partial regression coefficient ± SE | | | Intercept  ± SE | R^2^ | AIC | Δ AIC |
| --- | --- | --- | --- | --- | --- | --- | --- | --- |
|  |  | Log human population density | (Log human population density)^2^ | Logit (proportion land transformed) |  |  |  |  |
| 60 | 1 | -0.04 ± 0.04 | - | - | 0.07 ± 0.11 | 0.01 | 441.0 | 2.9 |
|  | 2 | -0.13 ± 0.06 | 0.03 ± 0.01 | - | -0.02 ± 0.11 | 0.04 | 438.1 | 0 |
|  | 3 | 0.08 ± 0.07 | - | -0.10 ± 0.05 | -0.43 ± 0.29 | 0.03 | 439.4 | 1.3 |
|  | 4 | - | - | -0.05 ± 0.03 | -0.14 ± 0.11 | 0.02 | 438.7 | 0.5 |
| 30 | 1 | 0.11 ± 0.02 | - | - | -0.18 ± 0.05 | 0.07 | 1496.6 | 0.5 |
|  | 2 | 0.10 ± 0.02 | 0.01 ± 0.01 | - | -0.21 ± 0.06 | 0.07 | 1497.7 | 1.6 |
|  | 3 | 0.08 ± 0.03 | - | 0.04 ± 0.02 | -0.01 ± 0.12 | 0.08 | 1496.1 | 0 |
|  | 4 | - | - | 0.09 ± 0.01 | 0.25 ± 0.06 | 0.07 | 1500.5 | 4.4 |
| 15 | 1 | 0.06 ± 0.01 | - | - | -0.09 ± 0.02 | 0.03 | 5334.7 | 36.5 |
|  | 2 | 0.07 ± 0.01 | -0.002 ± 0.003 | - | -0.08 ± 0.03 | 0.03 | 5336.2 | 37.9 |
|  | 3 | 0.004 ± 0.01 | - | 0.06 ± 0.01 | 0.16 ± 0.05 | 0.05 | 5300.2 | 1.9 |
|  | 4 | - | - | 0.06 ± 0.01 | 0.17 ± 0.03 | 0.05 | 5298.2 | 0 |
| 5 | 1 | 0.08 ± 0.003 | - | - | -0.06 ± 0.01 | 0.04 | 46861.9 | 246.4 |
|  | 2 | 0.08 ± 0.003 | -0.03x10^-2^ ± 0.001 | - | -0.06 ± 0.01 | 0.04 | 46863.8 | 248.3 |
|  | 3 | 0.03 ± 0.004 | - | 0.04 ± 0.002 | 0.13 ± 0.01 | 0.06 | 46615.5 | 0 |
|  | 4 | - | - | 0.05 ± 0.002 | 0.20 ± 0.01 | 0.05 | 46671.0 | 55.5 |
| 2 | 1 | 0.08 ± 0.001 | - | - | -0.04 ± 0.003 | 0.04 | 283799.0 | 1015.2 |
|  | 2 | 0.08 ± 0.001 | 0.001 ± 0.03x10^-2^ | - | -0.04 ± 0.003 | 0.04 | 283778.5 | 994.7 |
|  | 3 | 0.05 ± 0.002 | - | 0.02 ± 0.001 | 0.09 ± 0.01 | 0.05 | 282783.8 | 0 |
|  | 4 | - | - | 0.04 ± 0.001 | 0.18 ± 0.004 | 0.04 | 283636.3 | 852.5 |

**Table S5.** Partial regression coefficients (± standard errors; SE), coefficients of determination (R^2^) and AIC values, for four linear models relating species richness of butterflies with restricted ranges in southern Africa to human density and activity at five grid square scales (60, 30, 15, 5 and 2 minutes) within the extent of South Africa, using spatial Model 1: Linear model 1 – Restricted range species richness ~ Log human population density (number of people per 1 km^2^); Linear model 2 - Restricted range species richness ~ Log human population density + (Log human population density)^2^; Linear model 3 - Restricted range species richness ~ Log human population density + Logit proportion land transformed; Linear model 4 - Restricted range species richness ~ Logit proportion land transformed.

a)

| Grid scale | Linear model | Partial regression coefficient ± SE | | | Intercept  ± SE | R^2^ | AIC | Δ AIC |
| --- | --- | --- | --- | --- | --- | --- | --- | --- |
|  |  | Log human population density | (Log human population density)^2^ | Logit (proportion land transformed) |  |  |  |  |
| 60 | 1 | -0.03 ± 0.04 |  |  | 0.05 ± 0.11 | 0.004 | 441.5 | 1.2 |
|  | 2 | -0.04 ± 0.06 | 0.004 ± 0.01 |  | 0.04 ± 0.12 | 0.004 | 443.4 | 3.1 |
|  | 3 | 0.05 ± 0.07 |  | -0.07 ± 0.06 | -0.29 ± 0.29 | 0.01 | 441.8 | 1.4 |
|  | 4 |  |  | -0.04 ± 0.03 | -0.10 ± 0.11 | 0.01 | 440.3 | 0 |
| 30 | 1 | -0.01 ± 0.02 |  |  | 0.02 ± 0.05 | 0.04x10^-2^ | 1536.7 | 0 |
|  | 2 | 0.02 ± 0.03 | -0.01 ± 0.01 |  | 0.06 ± 0.06 | 0.004 | 1536.6 | 0 |
|  | 3 | -0.01 ± 0.03 |  | -0.02x10^-3^ ± 0.03 | 0.02 ± 0.12 | 0.04x10^-2^ | 1538.7 | 2.0 |
|  | 4 |  |  | -0.01 ± 0.01 | -0.01 ± 0.06 | 0.03x10^-2^ | 1536.7 | 0.1 |
| 15 | 1 | -0.003 ± 0.01 |  |  | 0.001 ± 0.02 | 0.01x10^-2^ | 5630.6 | 26.4 |
|  | 2 | 0.04 ± 0.01 | -0.02 ± 0.003 |  | 0.09 ± 0.03 | 0.02 | 5604.3 | 0 |
|  | 3 | -0.04 ± 0.01 |  | 0.03 ± 0.01 | 0.15 ± 0.05 | 0.01 | 5620.9 | 16.6 |
|  | 4 |  |  | 0.01 ± 0.01 | 0.04 ± 0.03 | 0.002 | 5627.0 | 22.7 |
| 5 | 1 | 0.01 ± 0.003 |  |  | 0.0003 ± 0.01 | 0.001 | 48402.8 | 111.1 |
|  | 2 | 0.03 ± 0.004 | -0.01 ± 0.001 |  | 0.05 ± 0.01 | 0.01 | 48291.8 | 0 |
|  | 3 | -0.01 ± 0.01 |  | 0.02 ± 0.003 | 0.09 ± 0.01 | 0.004 | 48355.0 | 63.2 |
|  | 4 |  |  | 0.01 ± 0.002 | 0.06 ± 0.01 | 0.004 | 48358.8 | 67.1 |
| 2 | 1 | 0.01 ± 0.001 |  |  | 0.004 ± 0.003 | 0.02x10^-2^ | 295587.6 | 324.0 |
|  | 2 | 0.01 ± 0.001 | -0.01 ± 0.03x10^-2^ |  | 0.03 ± 0.004 | 0.003 | 295263.7 | 0 |
|  | 3 | 0.02 ± 0.002 | -0.01 ± 0.001 |  | -0.06 ± 0.01 | 0.002 | 295371.3 | 107.6 |
|  | 4 | -0.01 ± 0.001 |  |  | -0.02 ± 0.004 | 0.001 | 295536.4 | 272.8 |

There was limited additional benefit of adding proportion of land transformed to the models (linear model 3; Tables S1, S2 and S5). Thus, the hypothesis that the impact of land transformation reduces richness (once shared causes of co-incidence are accounted for) had little or no support, as for Spatial Model 1.

**Supporting information S1: Figure legends**

**Figure S1. The correlation of butterfly species richness between two spatial models at five spatial resolutions.**

Scatter plots and correlation coefficients (r) of median butterfly species richness in South Africa between Spatial Model 1 (x-axis) and Spatial Model 2 (y-axis), at each grid square scale: a) 60 minutes, b) 30 minutes, c) 15 minutes, d) 5 minutes and e) 2 minutes. Dashed line indicates slope of one.

**Figure S2. South African human population density and proportion land transformed.**

(a) Human population density (log; number of people/km^2^) and (b) proportion land transformed (logit) in South Africa at the 2 minute grid square scale. High values of human population density and proportion of land transformed are represented by darker shades of grey, as shown by the keys.
